# IL-1 interacts with ethanol effects on GABAergic transmission in the mouse central amygdala

**DOI:** 10.3389/fphar.2015.00049

**Published:** 2015-03-19

**Authors:** Michal Bajo, Florence P. Varodayan, Samuel G. Madamba, Amanda J. Robert, Lindsey M. Casal, Christopher S. Oleata, George R. Siggins, Marisa Roberto

**Affiliations:** ^1^Committee on the Neurobiology of Addictive Disorders, The Scripps Research InstituteLa Jolla, CA, USA,; ^2^Molecular and Cellular Neuroscience Department, The Scripps Research InstituteLa Jolla, CA, USA

**Keywords:** IL-1β, central amygdala, GABA_A_, IPSCs, eIPSPs, interleukin, cytokine, IL-1ra

## Abstract

Neuroinflammation is hypothesized to enhance alcohol consumption and contribute to the development of alcoholism. GABAergic transmission in the central amygdala (CeA) plays an important role in the transition to alcohol dependence. Therefore, we studied the effects of interleukin-1β (IL-1β), a proinflammatory cytokine mediating ethanol-induced neuroinflammation, and its interaction with ethanol on CeA GABAegic transmission in B6129SF2/J mice. We also assessed ethanol intake in B6129SF2/J mice. Intake with unlimited (24 h) ethanol access was 9.2–12.7 g/kg (3–15% ethanol), while limited (2 h) access produced an intake of 4.1 ± 0.5 g/kg (15% ethanol). In our electrophysiology experiments, we found that recombinant IL-1β (50 and 100 ng/ml) significantly decreased the amplitude of evoked inhibitory postsynaptic potentials (eIPSPs), with no significant effects on paired-pulse facilitation (PPF). IL-1β (50 ng/ml) had dual effects on spontaneous miniature inhibitory postsynaptic currents (mIPSCs): increasing mIPSC frequencies in most CeA neurons, but decreasing both mIPSC frequencies and amplitudes in a few cells. The IL-1β receptor antagonist (IL-1ra; 100 ng/ml) also had dual effects on mIPSCs and prevented the actions of IL-1β on mIPSC frequencies. These results suggest that IL-1β can alter CeA GABAergic transmission at pre- and postsynaptic sites. Ethanol (44 mM) significantly increased eIPSP amplitudes, decreased PPFs, and increased mIPSC frequencies. IL-1β did not alter ethanol’s enhancement of the eIPSP amplitude, but, in IL-1β-responsive neurons, the ethanol effects on mIPSC frequencies were lost. Overall, our data suggest that the IL-1 system is involved in basal GABAergic transmission and that IL-1β interacts with the ethanol-induced facilitation of CeA GABAergic transmission.

## Introduction

Studies of human alcoholic brains and animal models have shown a link between the neuroimmune system and the brain changes associated with acute and chronic alcohol exposure (Crews and Vetreno, 2011; Crews et al., 2011; Harris and Blednov, 2012; Szabo et al., 2012; Szabo and Lippai, 2014). In particular, the interleukin-1 (IL-1) system has emerged as an important player in alcohol drinking and the development of alcohol dependence, and as a key regulator of alcohol-induced neuroimmune responses (Crews and Vetreno, 2011; Crews et al., 2011; Harris and Blednov, 2012; Szabo et al., 2012; Szabo and Lippai, 2014). The IL-1 system includes the cytokines IL-1α and IL-1β, the receptor IL-1R1, the IL-1R accessory protein (IL-1RAcP), and two negative regulators (a decoy receptor IL-1R2 and the IL-R1 antagonist: IL-1ra). The proinflammatory activities of the cytokines IL-1α and IL-1β are initiated by their binding to the IL-1 receptor (IL-1R1) and formation of a receptor heterodimeric complex with IL-1RAcP. After recruitment of the myeloid differentiation primary response gene 88 (MyD88) adaptor to the IL-1R/IL-1RAcpP complex, signaling pathways, including NF-κB, c-Jun N-terminal kinase (JNK) and p38 MAPK, are activated (Garlanda et al., 2013; Krumm et al., 2014).

Interleukin-1 and its receptor (IL-1R1) are expressed throughout the brain (Hagan et al., 1993; Ericsson et al., 1995; Quan et al., 1996, 1998; Taishi et al., 1997; Cartmell et al., 1999; French et al., 1999; Gayle et al., 1999; Parker et al., 2000; Hosoi et al., 2002; Johnson et al., 2004; Heida and Pittman, 2005) in both neurons (Allan et al., 2005) and glial cells (Blanco et al., 2005; Blanco and Guerri, 2007). Several studies have reported changes in the expression of genes encoding components of the IL-1R1 signaling pathways in the brains of mice with a genetic predisposition to alcohol consumption (Mulligan et al., 2006; Blednov et al., 2012). Additionally, polymorphisms in the genes encoding the IL-1R antagonist (IL-1ra; *Il1rn*) and IL-1β (*Il1b*), but not IL-1α (*Il1a*) and IL1-R1 (IL-1R1 type 1; *Il1r*), have been associated with a susceptibility to alcoholism or ALD (alcohol liver disease) in Spanish men (Pastor et al., 2005). Behavioral studies indicate a reduction in alcohol drinking and/or preference in *Il1rn* knockout mice (Blednov et al., 2012) and suggest an important role of the IL-1 system in alcohol’s effects. IL-1β levels are increased in alcoholics, as well as animal models of chronic alcohol exposure (Valles et al., 2004; Qin et al., 2008; Lippai et al., 2013a,b), and intracerebroventricular administrations of IL-1β potentiate alcohol withdrawal-induced anxiety (Breese et al., 2008). Conversely, administration of IL-1ra prevented and protected against alcohol-induced neuroinflammation (Lippai et al., 2013b), and reduced alcohol-induced sedation and motor impairment recovery time in mice (Wu et al., 2011).

As the central nucleus of the amygdala (CeA) plays a critical role in mediating alcohol-related and anxiety-like behaviors (Gilpin et al., 2014), it is likely that the IL-1 signaling system modulates ethanol’s effects on CeA function. In fact, we reported recently that the IL-1ra regulates baseline GABAergic transmission in the CeA and is critical for the effects of ethanol at these synapses (Bajo et al., 2014a). Additionally, immune challenges, such as systemic IL-1β or LPS administration, are known to activate the CeA (Dayas et al., 2001; Frost et al., 2001; Konsman et al., 2008). Moreover, IL-1R1 is expressed in the amygdala under basal conditions (Frost et al., 2001), while both IL-1β and IL-1ra are induced in the CeA by excitotoxic stimuli or systemic immune challenge (Eriksson et al., 2000; Konsman et al., 2008). This is particularly significant as the activation of IL-1R1 modulates synaptic transmission and plasticity (Zeise et al., 1992; Bellinger et al., 1995; Dunn et al., 1999; O’Connor and Coogan, 1999; Rothwell and Luheshi, 2000; Lin et al., 2006), glutamate and GABA release (Miller et al., 1991; Murray et al., 1997; Feleder et al., 1998; Sama et al., 2008; Mishra et al., 2012), and membrane expression of GABA receptors (Serantes et al., 2006; Wang et al., 2012).

As neuroinflammation plays an important role in alcohol use disorders and other psychiatric disorders (e.g., depression, PTSD; Jones and Thomsen, 2013), there are concerted efforts to develop new therapeutic strategies using compounds with anti-inflammatory properties to treat these disorders. Therefore, understanding the molecular and cellular mechanisms that mediate normal and pathological neuroimmune responses in the key brain regions involved in the pathogenesis of psychiatric disorders is critical for the evaluation of potential candidate drugs and their clinical use. Here, we examined the effects of IL-1β on GABAergic transmission in the CeA, as well as its actions on ethanol-induced facilitation of GABAergic transmission. We recorded from B6129SF2/J mice because they have been used previously as a control for *Il1r* KO mouse studies assessing the role of IL-1R1 in various biological phenomena ^1^ (for list of publications). Because alcohol-related behaviors in these mice have not been studied, we also characterized the B6129SF2/J strain for alcohol drinking and preference.

## Materials and Methods

### Animal Treatment

Male B6129SF2/J (*n* = 80; 29.5 ± 0.3 g) mice were housed in a temperature- and humidity-controlled room on a 12-h light/dark cycle (lights on at 6:00 pm) with food and water available *ad libitum*. We conducted all care procedures in accordance with the National Institutes of Health Guide for the Care and Use of Laboratory Animals and with the Institutional Animal Care and Use Committee policies of The Scripps Research Institute.

### Slice Preparation

The mice (10–16 weeks old at the time of electrophysiological recordings) were anesthetized with 3% isoflurane, decapitated, and the brains quickly removed and placed in ice-cold artificial cerebrospinal fluid (ACSF: composition in mM: NaCl, 130; KCl, 3.5; NaH_2_PO_4_, 1.25; MgSO_4_.7H_2_O, 1.5; CaCl_2_, 2.0; NaHCO_3_, 24; glucose, 10) and ice-cold oxygenated high-sucrose cutting solution (composition (in mM): sucrose, 206; KCl, 2.5; CaCl_2_, 0.5; MgCl_2_, 7; NaH_2_PO_4_, 1.2; NaHCO_3_, 26; glucose, 5; HEPES, 5; pH7.3–7.4) gassed with 95% O_2_ and 5% CO_2_. We cut coronal slices containing the CeA using a Leica 1000S vibratome cutter (Campden, Lafayette, IN, USA).

### Intracellular Recordings

We incubated the slices (400 μm) in an interface configuration for 30 min, and then completely submerged and continuously superfused (flow rate of 2–4 ml/min) them with warm (31^∘^C), O_2_/CO_2_-gassed ACSF. We added drugs to the ACSF from stock solutions to obtain known concentrations in the superfusate. We recorded from CeA neurons with sharp micropipettes containing 3 M KCl (65–80 MΩ resistance) using current-clamp mode. Data were acquired with an Axoclamp-2B preamplifier (now Molecular Devices, Sunnyvale, CA, USA) and stored for oﬄine analysis via pClamp 10.2 software (Molecular Devices). We evoked pharmacologically isolated GABA_A_ergic IPSPs by stimulating locally within the medial subdivision of the CeA with a bipolar stimulating electrode, while continuously superfusing the glutamate receptor blockers 6,7-dinitroquinoxaline-2,3-dione (DNQX, 20 μM) and DL-2-amino-5-phosphonopentanoic acid (DL-AP5, 30 μM), and the GABA_B_ blocker CGP 55845A (1 μM).

We held the CeA neurons near their resting membrane potentials (RMPs ranging from –65 to –85 mV (mean: –78.6 + 0.7 mV, *n* = 73), and applied hyperpolarizing and depolarizing current steps (200 pA increments, 750 ms duration) to generate voltage-current curves. To determine half-maximal IPSP amplitudes, we examined input/output (I/O) curves by measuring evoked IPSP amplitudes at five stimulus strengths ranging from the threshold to maximum stimulation. Subsequent analyses were done with averages of two IPSPs evoked with the half-maximal stimuli. We measured the IPSP amplitudes before (baseline), during (up to 20 min) and after (washout for 10–25 min) drug application, and determined the percent change in IPSP amplitude at each stimulus intensity using the equation: (Vdrug/Vcontrol) ^∗^ 100.

We examined paired-pulse facilitation (PPF) using 100 ms interstimulus intervals and the stimulus strength was adjusted so that the amplitude of the first IPSP was 50% of the maximal determined from the I/O relationship. We calculated the PPF using the equation: (2nd IPSP amplitude/1st IPSP amplitude) ^∗^ 100. We took PPF measurements before drug superfusion (baseline), during (10–20 min) and after drug washout (10–25 min).

### Whole-cell Patch-Clamp Recording

After cutting, the slices (300 μm) were incubated in O_2_/CO_2_-gassed ACSF for 30 min at 32^∘^C, followed by incubation for 30 min at room temperature. We performed whole-cell patch-clamp recording in voltage clamp mode, as described previously (Bajo et al., 2011). Briefly, we used infrared/DIC visualization of CeA neurons (Dodt and Zieglgansberger, 1990), followed by digitization and image enhancement via an upright, fixed-stage Olympus microscope. We used micropipettes with an input resistances of 3–6 MΩ (access resistance <20 MΩ, compensated 60–80%) filled with an internal solution (composition in mM: KCl, 145; EGTA, 5; MgCl_2_, 5; HEPES, 10; Na-ATP, 2; Na-GTP, 0.2; the latter two added fresh on the day of recording), pH 7.3–7.4. We isolated spontaneous miniature GABA_A_-mediated IPSCs (mIPSCs) pharmacologically by applying blockers of glutamatergic (20 μM DNQX, 30 μM DL-AP5) and GABA_B_ receptors (1 μM CGP 55845A), and adding 0.5 μM tetrodotoxin (TTX) to the bath. We used the Multiclamp 700B and pClamp 10.2 software (Molecular Devices) for data acquisition. Recombinant mouse IL-1β, recombinant IL-1ra and ethanol were added to the ACSF from stock solutions in known concentrations. We took all measures before drug (baseline) and during drug superfusion (12–15 min).

### Ethanol Drinking Procedure

This procedure was adapted from that of Blednov et al. (2005). Mice were allowed to acclimate for 1 week to individual housing. Two drinking tubes were continuously available, Monday–Friday, to each mouse and fluid consumption was measured daily. One bottle of water was available across weekends. Food was available *ad libitum* and mice were weighed each week. After 4 days of water consumption (on Monday, off Friday; water in both tubes), mice were offered 3% ethanol (v/v) versus water on the following Monday–Friday. We changed tube positions every day to control for position preferences. Over the following 4 weeks mice received 6, 9, 12, and 15% ethanol in this same manner. Following this, mice received 15% ethanol for 2 h per day (starting 3 h after lights off) for 5 days in order to examine limited access two bottle-choice (2BC) drinking. The quantity of ethanol consumed (g/kg body weight/24 h or 2 h) was calculated for each mouse and averaged across each 4–5 day measurement period.

### Data Analysis and Statistics

To analyze data acquired from intracellular and whole-cell recordings, we used Clampfit 10.2 (Molecular Devices) and MiniAnalysis 5.1 software (Synaptosoft, Leonia, NJ, USA), respectively. We used GraphPad Prism 5.0 (GraphPad Software, San Diego, CA, USA) software for all statistical analysis. We accepted statistical significance at the *p* < 0.05 level using one-way ANOVA and *t*-tests. The data are presented as percentile changes in mean ± SEM.

### Drugs

We purchased CGP 55845A, DNQX, and DL-AP5 from Tocris Biosciences (Ellisville, MI, USA), recombinant mouse IL-1β from Biolegend (San Diego, CA, USA), recombinant human IL-1ra from Peprotech (Rocky Hill, NJ, USA), and TTX from Calbiochem (San Diego, CA, USA). We obtained ethanol from Remet (La Mirada, CA, USA).

## Results

### Ethanol Drinking and Preference of B6129SF2/J Mice

**FIGURE 1:**
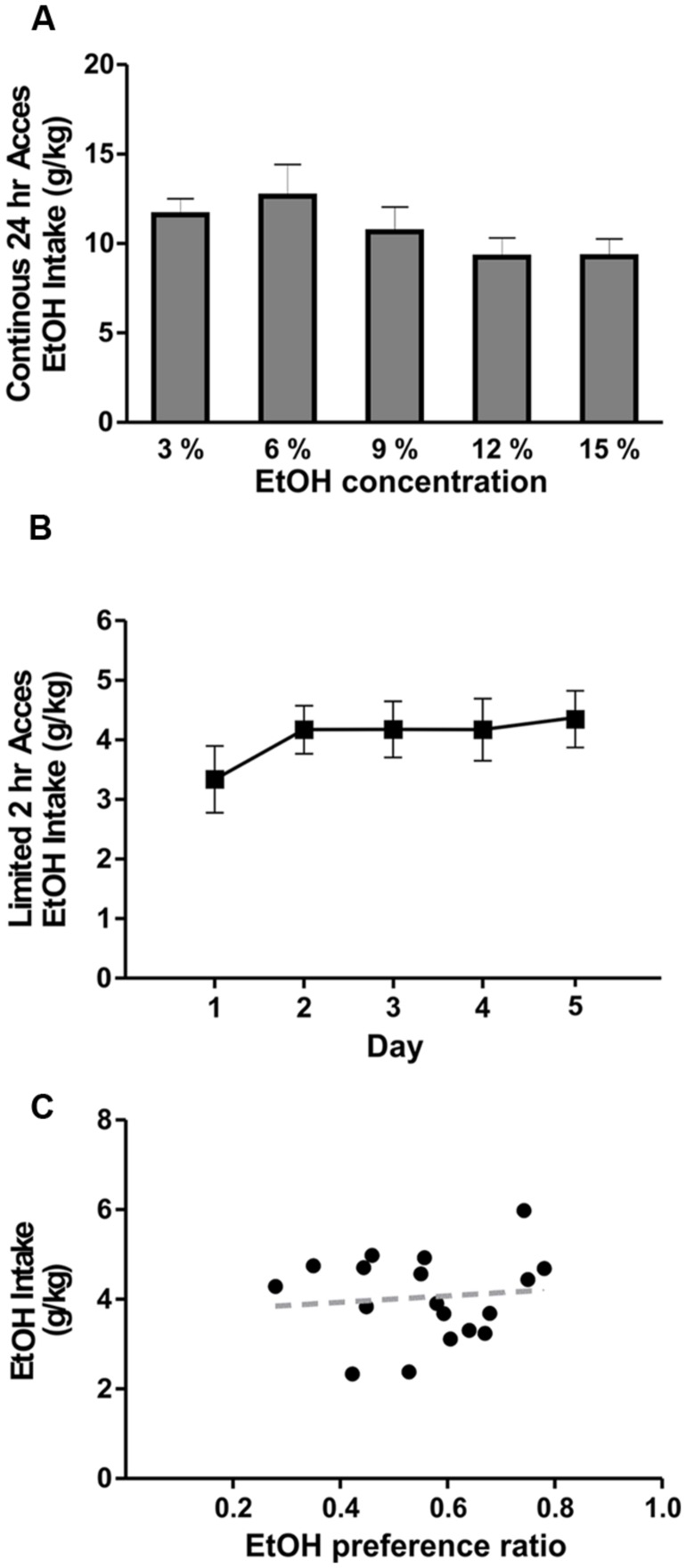
Ethanol drinking behavior of B6129SF2/J mice. The ethanol intake of B6129SF2/J mice was tested using unlimited (24 h) and limited (2 h) 2-bottle choice (2BC) paradigms. **(A)** The intake of 3, 6, 9, 12, and 15% ethanol was measured by 2BC with unlimited access to ethanol. On average, the mice consumed 11.6 ± 0.9 g/kg/day of 3% ethanol solution, 12.7 ± 1.8 g/kg/day of 6% ethanol solution, 10.7 ± 1.1 g/kg/day of 9% ethanol solution, 9.2 ± 1.1 g/kg/day at 12% ethanol solution, and 9.3 ± 1.0 g/kg/day of 15% ethanol solution. There was significant main difference in ethanol intake between the ethanol concentrations [*F*_(4,19)_ = 2.5, *n* = 20], but Tukey *post hoc* analysis did not reveal significant differences between specific ethanol concentrations. **(B)** For limited access measurements of ethanol consumption, we used 15% ethanol solution and intake was measured for 2 h daily (starting 3 h after lights off) for a period of 5 days. Consumption was 3.3 ± 0.6 g/kg of ethanol on day 1; 4.2 ± 0.4 g/kg of ethanol on day 2; 0.4.2 ± 0.5 g/kg on days 3 and 4; 4.4 ± 0.5 on day 5. Repeated measure one-way ANOVA showed no significant difference in ethanol intake between testing days [*F*_(4,17)_ = 0.7, *n* = 18]. **(C)** Ethanol intake of individual mice is plotted as a function of preference. The average ethanol preference ratio (volume of ethanol consumed/total volume of fluid consumed) was 0.56 ± 0.05. There was no significant correlation (*R*^2^ = 0.01) between ethanol preference and intake in B6129SF2/J mice.

Although the B6129SF2/J mice have been used previously as controls for *Il1r* KO mouse studies assessing the role of IL-1R1 in various biological phenomena, their ethanol drinking and preference behavior is unknown. Therefore, we used 2BC tests with unlimited (24 h) and limited (2 h) ethanol access to determine their voluntary drinking and preference. The average daily ethanol intake with 24 h access, measured for a period of 5 days, ranged from 9.24 to 12.65 g/kg for the ethanol concentrations tested (3, 6, 9, 12, and 15%; **Figure 1A**). There were no significant differences in ethanol intake between the tested concentrations, nor was there a correlation between the intake and ethanol concentration (*R*^2^= 0.7). During 2 h limited access, the average ethanol (concentration 15%) intake was 4.1 ± 0.5 g/kg (*n* = 18; **Figure B**) and the average ethanol preference ratio was 0.56 ± 0.05 (volume of ethanol consumed/total volume of fluid consumed). We did not observe a significant correlation between ethanol preference and ethanol intake (*R*^2^ = 0.01) in the individual B6129SF2/J mice (**Figure C**). These data indicate that B6129SF2/J mice drink a substantial amount of ethanol and have a modest preference for ethanol.

### IL-1β Decreased eIPSP Amplitudes in the CeA

**FIGURE 2:**
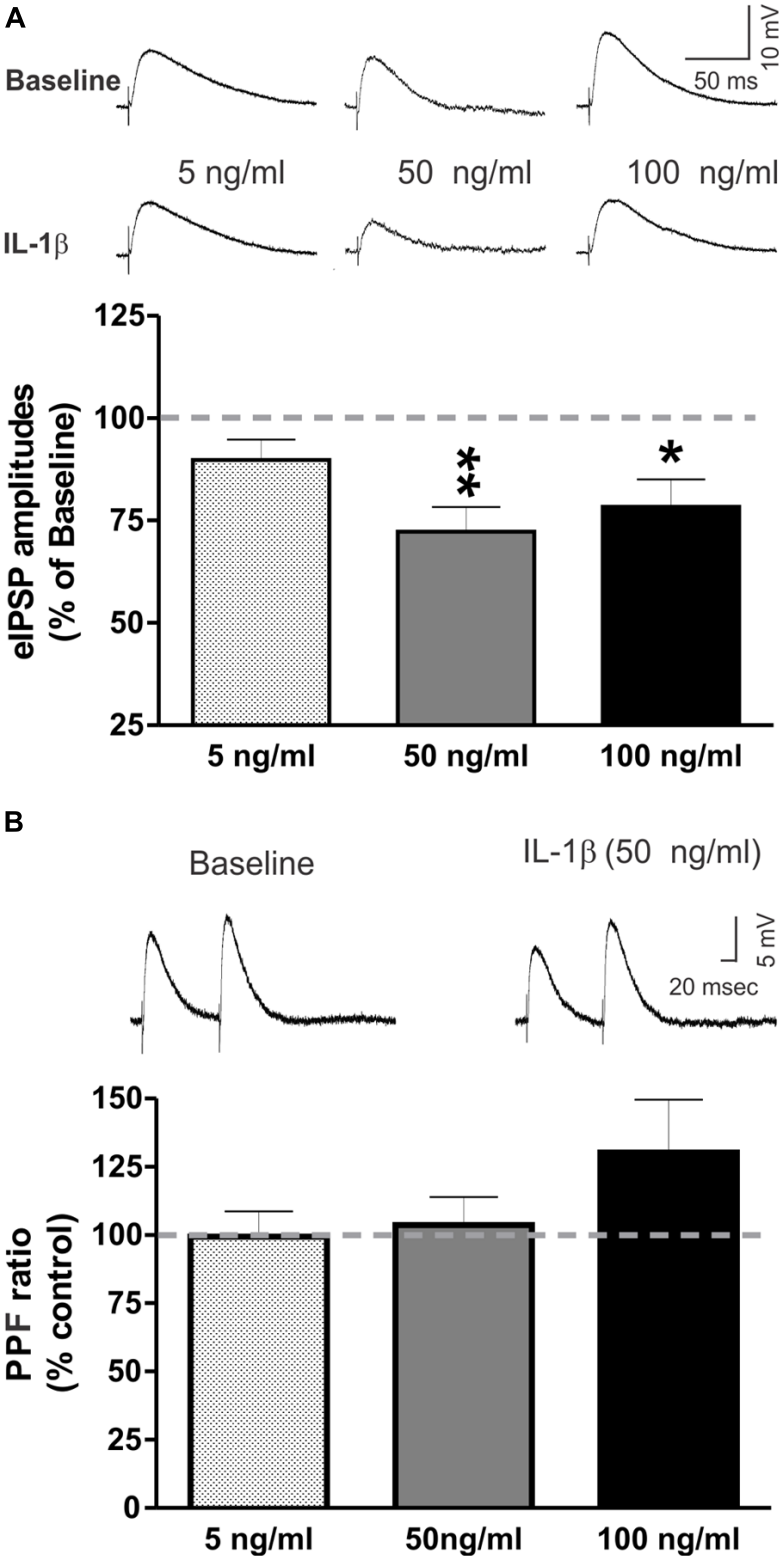
IL-1β decreases evoked GABA_A_-receptor mediated IPSPs in the CeA. **(A)** Recombinant mouse IL-1β decreased the mean amplitude of evoked IPSPs (eIPSPs) by 27.8 ± 6.0% (*n* = 11; *t*-test: *p* < 0.01) at 50 ng/ml and by 21.6 ± 6.7% (*n* = 5; *t*-test: *p* < 0.05) at 100 ng/ml. A lower concentration of IL-1β (5 ng/ml) had no significant effects on the eIPSPs (89.8 ± 5.0% of baseline, *n* = 7). (Top) Representative eIPSPs taken during baseline and IL-1β superfusion. (Bottom) Summary of the maximal effects of IL-1β elicited by the tested doses of IL-1β as compared to baseline. The statistical significance (^∗^*p* < 0.05 and ^∗∗^*p* < 0.01) was calculated by *t*-test. **(B)** IL-1β did not alter significantly paired-pulse facilitation (PPF; using a 100 ms interstimulus interval) at any of the tested concentrations (5 ng/ml: 99.4 ± 9.3% of baseline, *n* = 6; 50 ng/ml: 103.7 ± 7.2% of baseline, *n* = 8; 100 ng/ml: 130.1 ± 19.4% of baseline, *n* = 4). (Top) Representative recordings of PPF from a CeA neuron superfused with 50 ng/ml IL-1β. (Bottom) The PPF results are summarized on the bar graph with the PPF ratio during IL-1β superfusion compared to the baseline levels.

We tested the effects of recombinant mouse IL-1β (5, 50, and 100 ng/ml) on GABA_A_ receptor-mediated eIPSPs in the CeA. None of the tested concentrations significantly altered the current–voltage relationships, resting membrane potentials, or resistance (data not shown). In the majority of CeA neurons, high IL-1β concentrations (50 and 100 ng/ml) significantly decreased eIPSP amplitudes, by 27.8 ± 6.0% (*n* = 11; *t*-test: *p* < 0.01) and by 21.6 ± 6.7% (*n* = 5; *t*-test: *p* < 0.05), respectively (**Figure 2A**). However, 5 ng/ml IL-1β had no significant effect on the mean amplitudes of the eIPSPs (to 89.8 ± 5.0% of baseline, *n* = 7). The significant decreases in eIPSPs were not associated with changes in the PPF ratio, although there was a trend toward an increase in the PPF ratio by IL-1β at 100 ng/ml (50 ng/ml: 103.7 ± 7.2% of baseline, *n* = 8; 100 ng/ml: 130.1 ± 19.4% of baseline, *n* = 4; **Figure 2B**). Thus, these results indicate that IL-1β reduces GABAergic transmission, likely via postsynaptic mechanisms.

### IL-1β had Dual Effects on mIPSC Frequencies and Decreased mIPSC Amplitudes in CeA Neurons

**FIGURE 3:**
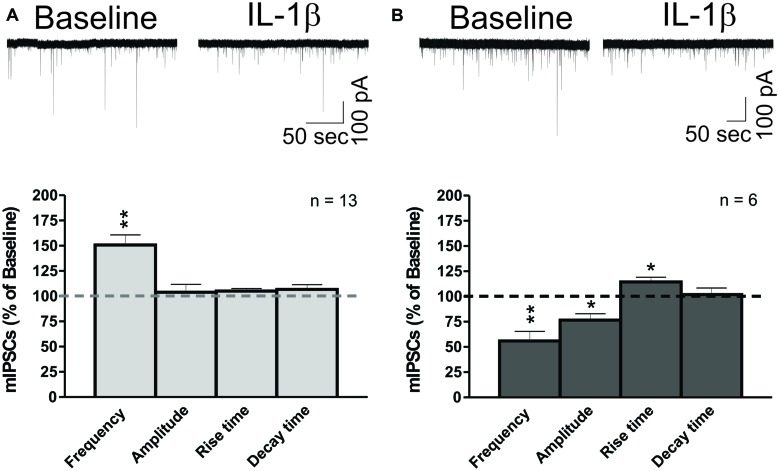
IL-1β has dual effects on miniature IPSCs in CeA neurons. **(A)** IL-1β increases mIPSC frequencies in the CeA. (Top) A representative whole- cell voltage clamp recording showing an increase in mIPSC frequency induced by 50 ng/ml IL-1β superfusion. (Bottom) The bar graphs present normalized mIPSC parameters from 62% of CeA neurons (13 of 21 cells) responding to IL-1β with an increase in mIPSC frequency (150.7 ± 10.0% of baseline). mIPSC amplitude (104.0 ± 7.8% of baseline) and kinetics were not significantly changed in these CeA neurons, although some cells showed individual changes in mIPSC amplitude. We calculated statistical significance (^∗∗^*p* < 0.01) by *t*-test. **(B)** IL-1β decreases mIPSC frequencies and amplitudes in CeA neurons. (Top) Representative recording of a CeA neuron responding to IL-1β with a decrease in mIPSC frequency. (Bottom) Acute application of IL-1β significantly decreased the mIPSC frequency (55.9 ± 9.5% of baseline) and amplitude (76.7 ± 6.2% of baseline) in 29% of CeA neurons (6 of 21 cells). In addition, IL-1β increased the mIPSC rise time (114.3 ± 4.7% of baseline) in these neurons. Statistical significance (^∗^*p* < 0.05) and (^∗∗^*p* < 0.01) were calculated by *t*-test.

We performed whole-cell recordings of mIPSCs in CeA neurons while superfusing 50 ng/ml of IL-1β. Here, we present the combined results of all experiments where IL-1β was applied to the naïve slice for 12–15 min (**Figure 3**). We found that IL-1β had dual effects (Δ > 15% from baseline) on mIPSC frequencies and amplitudes, and so we examined its effects on mIPSC frequencies and amplitudes separately. We found that IL-1β significantly increased the mean mIPSC frequency by 50.7 ± 10.1% in 13 of 21 CeA neurons (**Figure 3A**). In 6 of 21 cells, we observed a significant decrease in the mean mIPSC frequency by 44.1 ± 9.5%, which was associated with a significant decrease in the mean mIPSC amplitude (76.7 ± 6.2% of baseline) and an increase in mIPSC rise time (114.4 ± 4.7% of baseline; **Figure 3B**). In the remaining 2 neurons, IL-1β had no effect on mIPSC frequency (data not shown). Using the changes in mIPSC amplitude as the parameter for the division of the data, we found a significant decrease in the mIPSC amplitude in 8 of 21 cells (72.2 ± 6.2% of baseline) and no effects in 10 of 21 cells. In the remaining three CeA neurons, IL-1β increased both the mIPSC amplitude by 46.0 ± 14.7% and frequency by 62.8 ± 26.7% (data not shown). Since changes in mIPSC frequencies suggest an altered probability of vesicular transmitter release, and changes in mIPSC amplitudes may reflect modulation of postsynaptic GABA_A_ receptors (De Koninck and Mody, 1994; Otis et al., 1994), our data indicate that IL-1β alters spontaneous action potential-independent GABA transmission through both presynaptic and postsynaptic mechanisms of action. Importantly, the parallel changes in mIPSC frequencies and amplitudes of individual CeA neurons suggest that acute IL-1β acts in a cell-specific manner.

### IL-1ra Modulates CeA mIPSCs and Blocks the Effects of IL-1β on mIPSCs

**FIGURE 4:**
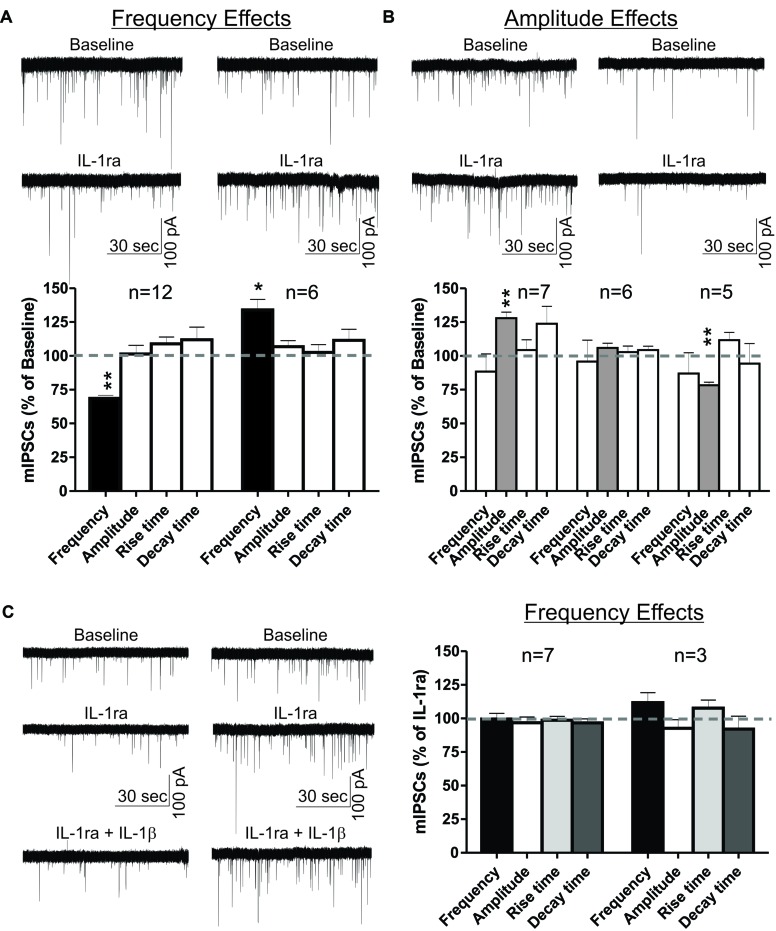
IL-1ra has dual effects on basal mIPSCs and prevents IL-1β-induced modulation of mIPSCs in the CeA. IL-1ra-induced changes in mIPSC frequencies and/or amplitudes vary among CeA neurons, indicating cell-specific differences in IL-1ra modulation of GABAergic transmission. **(A)** Dual IL-1ra-induced changes in mIPSC frequencies. (Top) Representative recordings of two CeA neurons showing a decrease (left column) or increase (right column) in mIPSC frequencies following IL-1ra application (100 ng/ml). (Bottom) Summary bar graph showing IL-1ra decreased significantly (*t*-test, *p* < 0.01) the mean mIPSC frequency by 31.3 ± 2.1% in 12 of 18 (67%) neurons. In the remaining CeA cells (6 of 18), IL-1ra increased the mIPSC frequency by 34.1 ± 7.7% (*t*-test, *p* < 0.05). The changes in mIPSC frequencies were not associated with significant changes in mIPSC amplitudes or kinetics. **(B)** The IL-1ra induced changes in the mIPSC amplitudes were also variable. (Top) Representative recordings of two cells responding to IL-1ra with increased (left column) or decreased (right column) mIPSC amplitudes. (Bottom) Summary bar graph showing IL-1ra increased (*t*-test, *p* < 0.01) mIPSC amplitudes by 27.9 ± 4.5% in 7 of 18 cells (39%) and decreased (*t*-test, *p* < 0.01) by 21.7 ± 5.9% in 5 of 18 cells (28%). There were no significant changes in the mean mIPSC frequencies and kinetics across all cell groups. The statistical significance (^∗^*p* < 0.05) and (^∗∗^*p* < 0.01) was calculated by *t*-test. **(C)** To examine the effects of IL-1ra on the IL-1β-induced modulation of mIPSCs, we compared the mIPSC parameters recorded within 9–15 min of 100 ng/ml IL-1ra and 50 ng/ml IL-1β co-application to the last 6 min (9–15 min) of IL-1ra application alone. We divided the CeA neurons into two groups according to their cellular responses (mIPSCs frequency) to IL-1ra alone: the cells that responded to IL-1ra with decreased mIPSC frequency [by 31.8 ± 3%; *F*_(2,23)_ = 7.5, *p* < 0.05; *n* = 7] and the cells that responded to IL-1ra with increased mIPSC frequency [by 27.9 ± 7%; *F*_(2,8)_ = 1.7, *p* < 0.05; *n* = 3]. (Left) Representative recordings from two CeA neurons responding to IL-1ra with decreased (left column) or increased (right column) mIPSCs frequencies. (Right) IL-1ra prevented the IL-1β-induced modulation of mIPSCs, as there were no significance differences in mIPSCs after co-application of IL-1ra and IL-1β compared to IL-1ra alone. The statistical significance was set at (^∗^*p* < 0.05) and was calculated by repeated measurement one-way ANOVA followed by a Tukey* post hoc* test.

To examine the role of IL-1R1 in the effects of IL-1β in the CeA, we used an IL-1R1 antagonist (recombinant IL-1ra) to block IL-1β’s actions on GABAergic transmission. Here, we present the results of experiments where IL-1ra (100 ng/ml) was applied to the naïve slice for 12–15 min, and the subset of these experiments where IL-1β was subsequently co-applied for 12–15 min (**Figure 4**). We observed transient IL-1ra effects with maximal cellular responses within 9–15 min of drug application. Similar to the IL-1β effects, the IL-1ra-induced changes in mIPSC frequency and/or amplitude varied among individual CeA neurons. In the majority (67%) of CeA cells, IL-1ra decreased significantly the mean mIPSC frequency by 31.3 ± 2.1% (**Figure 4A**). In the remaining cells, IL-1ra significantly increased the mIPSC frequency by 34.1 ± 7.7% (**Figure 4B**). These changes in the mIPSC frequencies were not associated with significant changes in mIPSC amplitudes or kinetics. On the other hand, when we used the change in mIPSC amplitude (Δ > 15%) as the criterion for cell grouping, we found that IL-1ra increased significantly the mean mIPSC amplitude by 27.9 ± 4.5% in 39% of the cells and decreased the mean mIPSC amplitude by 21.7 ± 5.9% in 28% of CeA neurons (**Figure 4B**). These changes in mIPSC amplitudes were not associated with significant changes in mIPSC frequencies or kinetics. In the rest of the cells (33%), IL-1ra did not alter significantly the mean mIPSC frequency, amplitude or kinetics. These results indicate that IL-1R1 plays a role in basal GABAergic transmission in the CeA.

We also examined whether IL-1ra prevents the IL-1β-induced changes in mIPSCs. In order to do this, we grouped the neurons into two groups based on their IL-1ra-induced changes in mIPSC frequencies, and compared their mean mIPSC characteristics to the average mIPSC characteristics observed after 12–15 min of IL-1ra and IL-1β co-application. Co-application with IL-1β did not induce significant differences in the mIPSCs compared to IL-1ra alone (**Figure 4C**). These results suggest that IL-1β modulates mIPSCs via IL-1R1.

### Ethanol Increased eIPSPs and mIPSCs in the CeA Via a Predominantly Presynaptic Mechanism

**FIGURE 5:**
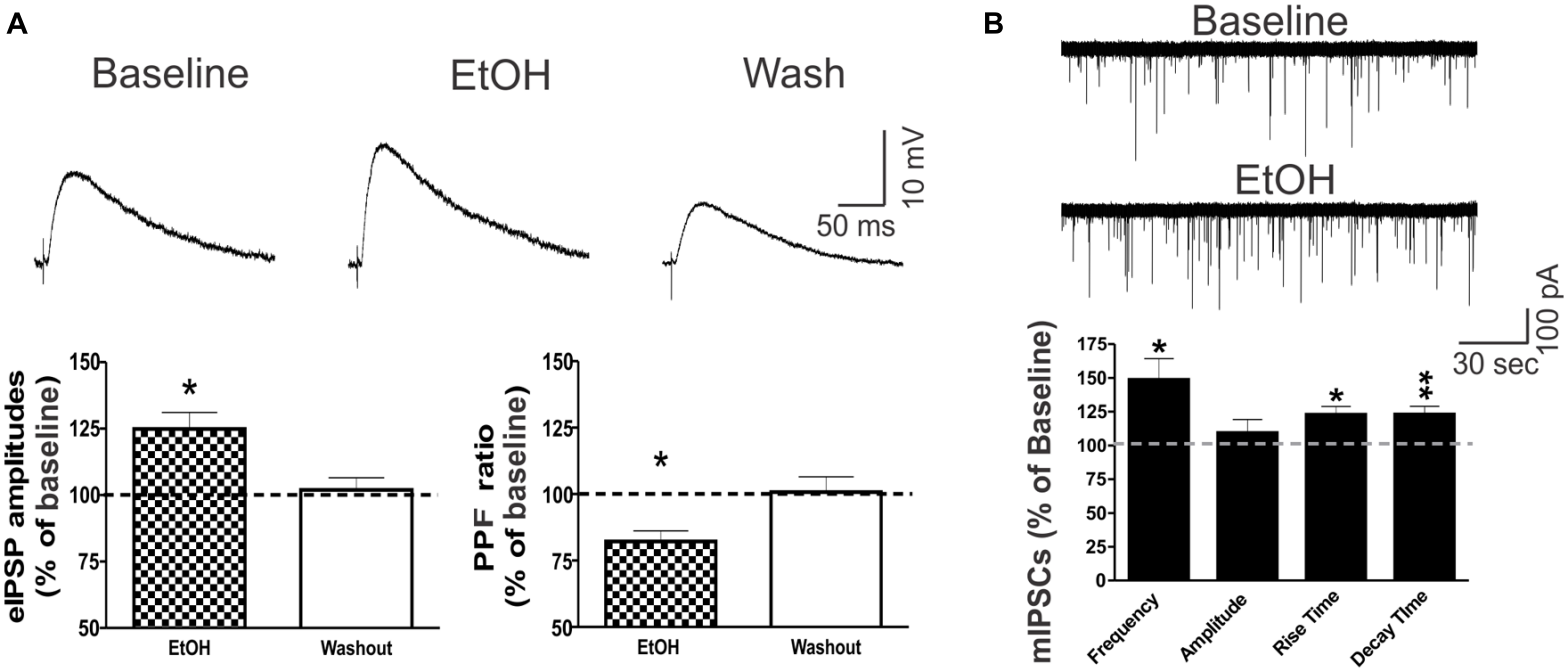
Ethanol potentiates CeA GABAergic transmission. **(A)** Ethanol potentiated eIPSPs via a presynaptic mechanism in CeA neurons. (Top) Representative recordings of eIPSPs from a CeA neuron showing an ethanol-induced increase in eIPSP amplitude that is reversed upon drug washout. (Bottom) On average, 44 mM ethanol significantly increased the mean eIPSP amplitude by 22.5 ± 5.9% (left column: *n* = 8; *t*-test: *p* < 0.05) and decreased the PPF ratio to 82.2 ± 4.0% of baseline in six of eight neurons (right column: *n* = 6; *t*-test: *p* < 0.05), indicating that ethanol-induced eIPSP potentiation is mediated by increased GABA release. **(B)** Ethanol increases spontaneous miniature GABA transmission in the CeA by both pre- and postsynaptic mechanisms. (Top) Representative mIPSC recordings from a CeA neuron showing an ethanol-induced increase in frequency. (Bottom) Superfusion of 44 mM ethanol induced a significant increase in the mean mIPSC frequency (140.7 ± 17.5% of baseline), but had no effect on the mean amplitude (102.6 ± 9.5% of baseline; *n* = 4, *t*-test: *p* < 0.05), supporting the finding that ethanol’s mechanism of action is predominantly presynaptic. However, ethanol significantly altered mIPSC kinetics, with a 14.7 ± 4.2% increase in the rise time and a 25.4 ± 7.0% increase in the decay time, indicating additional postsynaptic changes (*t*-test: ^∗^*p* < 0.05 and ^∗∗^*p* < 0.01).

Ethanol (44 mM) had no significant effects on the intrinsic membrane properties (resting membrane potential, the current–voltage relationship, resistance) of CeA neurons (data not shown; see also Roberto et al., 2003). Superfusion of 44 mM ethanol increased the mean eIPSP amplitude by 22.5 ± 5.9% in CeA neurons and significantly decreased the PPF ratio (82.2 ± 4.0% of baseline), suggesting that ethanol acts via presynaptic mechanisms (**Figure 5A**). This finding is supported by ethanol’s facilitation of the mean mIPSC frequency by 40.7 ± 17.5% (**Figure 5B**). Although ethanol had no effect on the mean mIPSC amplitude, it significantly increased the mIPSC rise (by 14.7 ± 4.2%) and decay (by 25.4 ± 7.0%) times.

### Co-Application of Ethanol Reversed the IL-1β-Induced Decrease in the Mean eIPSP Amplitude

**FIGURE 6:**
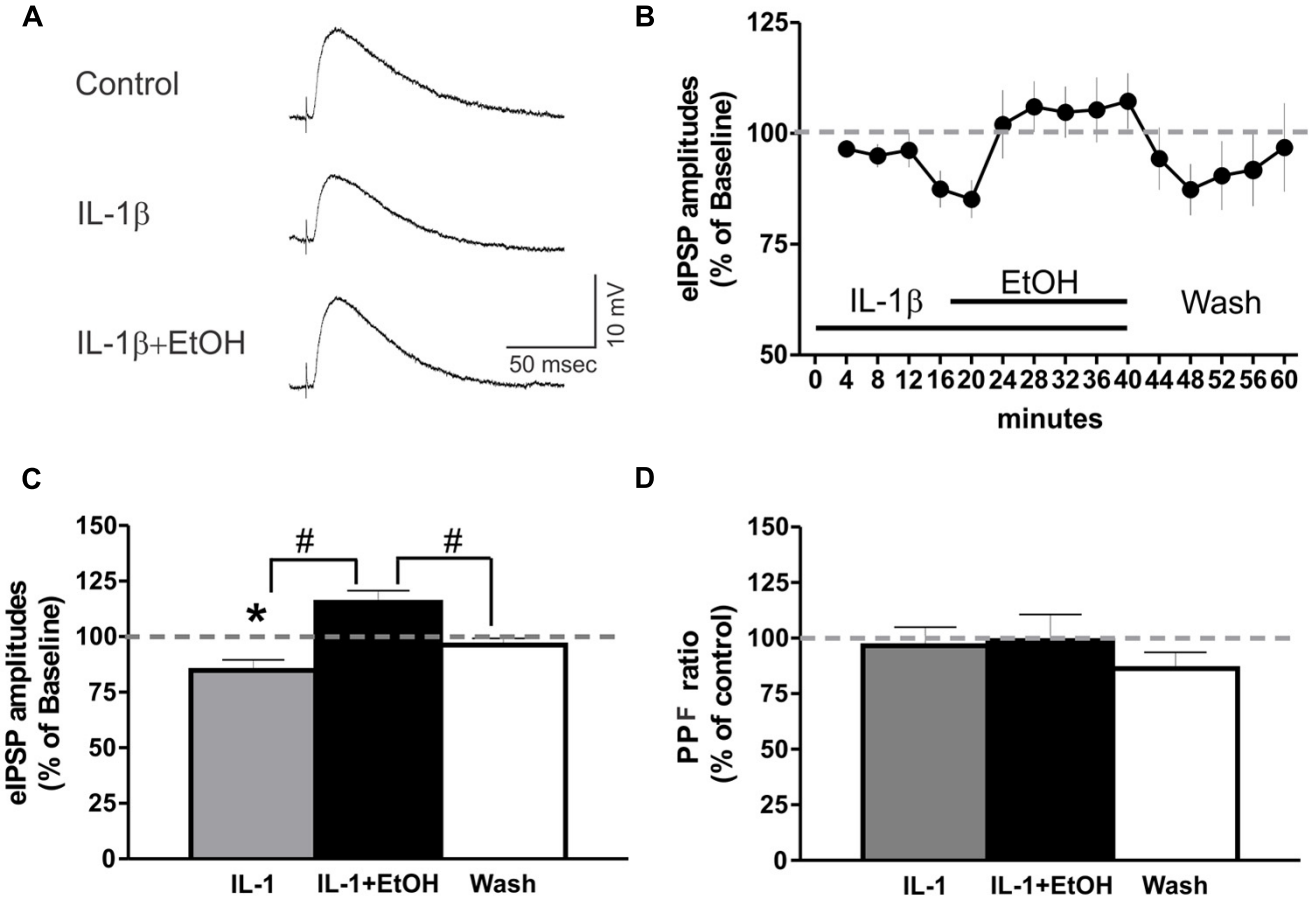
IL-1β and ethanol have opposing effects on eIPSP amplitudes. **(A)** Representative eIPSPs from a CeA neuron showing a 50 ng/ml IL-1β-induced decrease in eIPSP amplitude, and its subsequent reversal to baseline levels by the addition of ethanol (44 mM). **(B)** Time course presenting the averaged eIPSP amplitudes over 3 min bin periods. **(C)** Co-application of ethanol reversed the IL-1β-induced decrease in mean eIPSP amplitude [84.8 ± 4.7% of baseline, *n* = 9; *F*_(2,26)_ = 12.1, *p* < 0.01] to slightly above baseline levels (115.4 ± 5.3% of baseline). Statistical significance [*p* < 0.05; ^∗^(comparisons to baseline) and ^#^(comparison of the effects of ethanol plus IL-1β co-application to IL-1β alone or washout)] was calculated by repeated measurement one-way ANOVA followed by a Tukey *post hoc* test. **(D)** There were no significant effects on the PPF ratio (100 ms interstimulus interval) of IL-1β alone, or when it was co-applied with ethanol [*F*_(2,23)_ = 0.24].

We then examined the interaction between IL-1β and ethanol on GABAergic transmission in the CeA, by superfusing IL-1β (50 ng/ml) for 15–20 min, followed by co-application of IL-1β and ethanol (44 mM) for an additional 15–20 min. Using intracellular recording, we found no significant changes in the membrane properties induced by IL-1β or co-application of IL-1β and ethanol (data not shown). IL-1β alone significantly decreased the mean eIPSP amplitude (84.8 ± 4.7% of baseline), whereas co-application with ethanol reversed the IL-1β-induced decrease in the mean eIPSP amplitude back to 115.4 ± 5.3% of the original baseline (**Figure 6A,B**). Ethanol co-application significantly increased the mean eIPSP amplitude in comparison to the IL-1β effect, but not with respect to the baseline level (**Figure 6C**). In addition, we did not observe significant changes in the PPF ratio following superfusion with IL-1β alone or co-application of IL-1β and ethanol (**Figure 6D**). These results indicate that IL-1β and ethanol modulate CeA eIPSPs via different mechanisms, though the occlusion of ethanol’s PPF effects suggest that IL-1β may interfere with the downstream mechanisms mediating ethanol-facilitated GABA release.

### IL-1β Occluded Ethanol Effects on CeA mIPSCs

**FIGURE 7:**
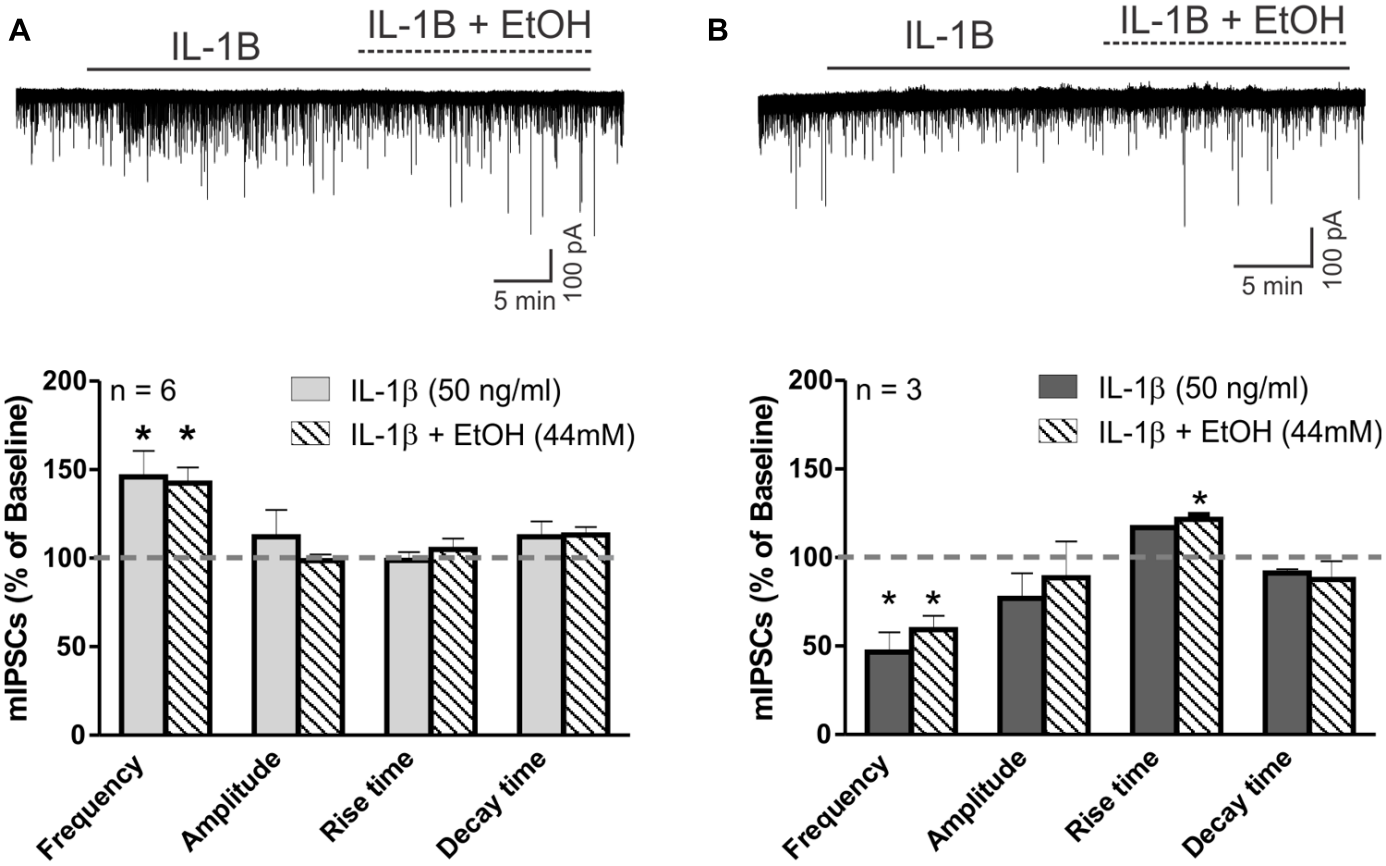
IL-1β occludes ethanol’s facilitation of mIPSCs. **(A)** The effects of ethanol on mIPSCs are blocked by IL-1β in cells that previously showed increased mIPSC frequency in the presence of IL-1β alone. (Top) Voltage clamp recordings of mIPSCs from a CeA neuron showing an IL-1β-induced increase in mIPSC frequency that is unaltered by the addition of ethanol. (Bottom) Summary of the normalized mIPSC maximal effects of IL-1β (50 ng/ml) alone, and IL-1β and ethanol (44 mM) co-application. IL-1β alone significantly increases mIPSC frequency by 45.9 ± 14.6% in 6 of 10 CeA neurons. The co-application of ethanol did not further change the mIPSC frequency (142.4 ± 8.9% of baseline; [*F*_(2,5)_ = 6.9, *p* < 0.05; Tukey *post hoc* test]. There were no differences in mIPSC amplitudes and kinetics across all treatments. Statistical significance (^∗^*p* < 0.05) was calculated by one-way ANOVA followed by a Tukey* post hoc* test. **(B)** Ethanol’s effects on mIPSC frequency are blocked by IL-1β in cells that previously showed decreased mIPSC frequency in the presence of IL-1β alone. (Top) Representative recordings from a CeA neuron showing a reduction in mIPSC frequency elicited by IL-1β and the co-application of IL-1β and ethanol. (Bottom) In 3 of 10 CeA neurons, mIPSC frequency was significantly decreased by IL-1β (46.5 ± 11.3% of baseline) alone, as well as with the co-application of IL-1β and ethanol [59.2 ± 7.9% of baseline; *F*_(2,2)_ = 35.5, *p* < 0.05; Tukey *post hoc* test]. There was no significant difference between the effects of IL-1β alone and co-application of IL-1β and ethanol (Tukey *post hoc* test, *p* < 0.05), but co-application of IL-1β and ethanol significantly increased the mean rise time of mIPSCs [116.4 ± 5.4% of baseline; *F*_(2,2)_ = 7.9, *p* < 0.05; Tukey *post hoc* test]. The statistical significance (^∗^*p* < 0.05) was calculated by one-way ANOVA followed by a Tukey* post hoc* test.

Finally, we investigated the potential interaction between IL-1β and ethanol on action potential-independent vesicular GABA release. In the majority of CeA neurons (6 of 10 cells), IL-1β alone, as well as its co-application with ethanol, significantly increased the mean mIPSC frequency (to 145.9 ± 14.6% and 142.4 ± 8.9% compared to baseline, respectively; **Figure 7A**). There were no significant changes in the mean mIPSC amplitudes or kinetics with IL-1β or co-application of IL-1β and ethanol in these cells (**Figure 7A**). However, in 3 of 10 cells, IL-1β alone decreased the mIPSC frequency by 53.5 ± 11.3%, and subsequent co-application of ethanol did not alter this IL-1β-induced decrease in mIPSC frequency (remained at 59.2 ± 7.9% of baseline; **Figure 7B**). Although there was a trend toward a decrease in mIPSC amplitudes by IL-1β and co-application of IL-1β and ethanol (82.15 ± 9.7% and 88.14 ± 11.8 of baseline, respectively) in these three neurons, it did not reach statistical significance. Finally, there was one CeA cell that showed no IL-1β effect on mIPSC frequency, but co-application of ethanol increased the mIPSC frequency to 127.6% of baseline. In this neuron, the mIPSC amplitude was decreased by IL-1β alone (by 30.8%) and also by ethanol co-application (by 32.1%).

## Discussion

In the present study, we investigated cytokine IL-1β modulation of GABAergic transmission and its interaction with ethanol-induced facilitation of GABA signaling in the CeA of B6129SF2/J mice. Behaviorally, B6129SF2/J mice have a moderate preference for alcohol and consume a substantial amount of alcohol. At the cellular level, IL-1β modulation of CeA GABAergic transmission is characterized by a reduction of evoked IPSPs, mediated predominantly by postsynaptic mechanisms, and by predominantly presynaptic dual effects on spontaneous miniature IPSCs in a cell-specific manner. The IL-1β effects on mIPSCs appear to be mediated by IL-1R1. Moreover, IL-1R1 regulates basal mIPSCs in the CeA. The interaction of IL-1β and ethanol is likely to occur presynaptically, and is characterized by the occlusion of ethanol’s facilitation of vesicular GABA release by IL-1β.

B6129SF2/J mice have been used previously as controls for *Il1r* KO mice (#003018, Jackson Laboratories) in studies characterizing the role of IL-1R1 in various physiological and pathological processes ^2^ (for a list of publications). Alcohol drinking behavior in B6129SF2/J mice has not been determined, despite the fact that different mice strains exhibit a range of alcohol drinking behaviors in terms of alcohol consumption and preference (Rhodes et al., 2007; Yoneyama et al., 2008). Since the genetic background of B6129SF2/J mice is based on C57BL/6J and 129S1/SvImJ mice, we expected to find similarities in the alcohol drinking phenotype of B6129SF2/J mice to those two strains, particularly the C57BL/6J mice. Our behavioral data showed that ethanol intake and ethanol preference of B6129SF2/J mice are similar to the values reported for C57BL/6J mice (Yoneyama et al., 2008).

Cytokines, including IL-1, play an important role in the regulation of both excitatory and inhibitory neurotransmission in the central nervous system (Camacho-Arroyo et al., 2009). IL-1R1 is expressed on glial cells and neurons, and thus, the overall effect of IL-1 on synaptic transmission is a combination of the direct effects of IL-1 binding to neuronal IL-1R1 and the indirect effects mediated by other signaling molecules generated and released by both neurons and glia in response to IL-1/IL-1R1 binding (e.g., cytokines, chemokines, ATP, etc.). The IL-1β effects on GABAergic transmission appear to be brain region specific, as IL-1 increases GABAergic transmission in some regions (e.g. hypothalamus, hippocampus; Miller et al., 1991; Plata-Salaman et al., 1998; Tabarean et al., 2006) and decreases it in others (e.g., basolateral amygdala, cerebellum; Yu and Shinnick-Gallagher, 1994; Pringle et al., 1996).

In our study we determined a concentration response curve for IL-1β, and found that only higher concentrations of IL-1β (>5 ng/ml) were effective in the modulation of CeA GABAergic transmission. The fact that the effective doses in our study are higher than in other brain regions (often in pg/ml range) may be caused by regional differences in the IL-1 system, especially in the expression of IL-1R1 (Wong and Licinio, 1994; Yabuuchi et al., 1994; Ericsson et al., 1995). To examine the role of IL-1R1 in the IL-1β effects, we used a recombinant IL-1R1 antagonist (IL-1ra). IL-1ra blocks the effects of IL-1β on mIPSCs, indicating that IL-1R1 mediates the IL-1β-induced modulation of CeA GABAergic transmission. We also observed a transient modulation of mIPSCs by IL-1ra alone, indicating that IL-1R1 regulates basal mIPSCs. In agreement with this finding, we reported recently an important role of IL-1ra and the IL-1 system in basal CeA GABAergic transmission. In that study, we observed an increase in the frequency of the spontaneous action potential-dependent IPSCs in IL-1ra deficient mice, but mISPC frequencies (action potential-independent IPSCs) were not affected (Bajo et al., 2014a). In addition to the compensatory mechanism associated with knockout technology and the different strains of mice used in the two studies (B6129SF2/J vs. C57Bl6J), the transiency of the IL-1ra effects on mIPSC in the current study may explain the lack of differences between baseline mIPSC frequencies of IL-1ra deficient mice and wild-type controls. Overall, both studies indicate that IL-1ra and the IL-1 system are involved in the regulation of basal GABAergic transmission in the mouse CeA. In the hippocampus, IL-1R1 also plays a critical role in baseline neuronal activity, (Hellstrom et al., 2005), while IL-1ra alone had no effects in neurons from the paraventricular nucleus of the hypothalamus (Ferri and Ferguson, 2003) or the spinal cord (Liu et al., 2013). Collectively, these findings further support that the regional specificity of the IL-1 system-dependent regulation of neuronal activities may underlie the brain region differences in the neuropathology associated with neuroinflammation.

Additionally, in our study, the IL-1β and IL-1ra effects on GABAergic transmission occurred in a majority of CeA neurons, and the effects were characterized by a duality of responses in individual CeA neurons. Other groups have reported a similar duality in their results, with electrophysiological studies revealing that IL-1β only affects synaptic transmission in a portion of neurons in the amygdala, cerebellum, hippocampus, and hypothalamus (Miller et al., 1991; Yu and Shinnick-Gallagher, 1994; Pringle et al., 1996; Plata-Salaman et al., 1998; Tabarean et al., 2006). It is therefore plausible to speculate that the cell-specific IL-1β and IL-1ra effects that we observed are determined by neuronal type, especially given the considerable heterogeneity of CeA neurons in terms of their biochemical and electrophysiological properties (Chieng et al., 2006; Herman et al., 2013) and their likely IL-1R1 expression. We classified each CeA neuron according to the electrophysiological criteria used in our previous studies on cell-type specific tonic GABA conductance in the CeA (Herman et al., 2013; Herman and Roberto, 2014), but did not observe any correlation between the IL-1β or IL-1ra effects and cell-type (low-threshold bursting, late spiking and regular spiking CeA neurons). Instead, it is likely that the cell-specificity of the IL-1β and IL-1ra effects are determined by the CeA neuronal expression of IL-1R1 (pre- versus postsynaptic expression), signaling pathways and/or other biochemical properties. Although our findings strongly indicate that the IL-1β effects on GABAergic transmission are mediated by IL-1R1, we cannot rule out completely that some of the IL-1β effects in the CeA may be caused by indirect actions of IL-1β via other signaling molecules (Camacho-Arroyo et al., 2009).

In this study, we have corroborated in B6129SF2/J mice our previous findings on ethanol’s facilitation of GABAergic transmission in the mouse CeA (mostly C57BL6/J; Bajo et al., 2008, 2014b; Kang-Park et al., 2009; Cruz et al., 2011; Herman et al., 2013). In CeA slices from B6129SF2/J mice, ethanol potentiated both evoked and spontaneous CeA GABAergic transmission predominantly via presynaptic mechanisms, but also had limited postsynaptic effects. To investigate the potential interaction between ethanol and IL-1β, we pretreated slices with IL-1β, and then co-applied ethanol and IL-1β. Ethanol, in the presence of IL-1β, was still able to potentiate evoked GABAergic transmission, despite the IL-1β-induced reduction in evoked IPSP amplitudes. However, the effects of ethanol co-application with IL-1β were not significantly different when compared to the original baseline levels. These data suggest that the mechanisms of action of IL-1β and ethanol on evoked GABA transmission are different, in line with our findings that IL-1β acts via predominantly postsynaptic mechanisms, whereas ethanol acts presynaptically. In the case of spontaneous GABAergic transmission in the CeA, IL-1β has dual effects, either increasing or decreasing vesicular GABA release. Notably, ethanol co-application with IL-1β did not facilitate further vesicular GABA release in the neurons that had previously responded to IL-1β with an increase in mIPSC frequency. Ethanol also failed to increase GABA release in the neurons that responded to IL-1β with decreased mIPSC frequency, suggesting an occlusion of ethanol’s effects by IL-1β pretreatment. The differences in eIPSP and mIPSC findings on the interaction of IL-1β and ethanol are likely to originate from differences in the forms of GABA release involved in each kind of synaptic transmission (Mathew et al., 2008; Fredj and Burrone, 2009). Specifically, mIPSCs are recorded in the presence of TTX to block Na^+^ channels and consequently, the generation of action potentials. In contrast, evoked IPSPs require stimulation of a synaptic network, and thus, action potential-dependent release (Farrant and Nusser, 2005).

## Conclusion

Our data collectively demonstrate that B6129SF2/J mice show an ethanol phenotype similar to that of C57BL6J mice, both behaviorally and electrophysiologically, in the CeA. IL-1β modulation of CeA GABAergic transmission is complex and characterized by dual and cell-specific modulations of presynaptic GABA release and postsynaptic GABA_A_ receptor activity. With regard to the IL-1β effects on ethanol-induced facilitation of CeA GABAergic transmission, our data indicate that IL-1β interacts with ethanol presynaptically to occlude ethanol’s enhancement of GABA signaling. Understanding these complex interactions of acute ethanol with IL-1 on GABAergic transmission are critical for shedding light on the potential role of the IL-1 neuroimmune system in the development of alcohol dependence and addiction.

## Footnotes

1 http://jaxmice.jax.org/strain/003018.html

2 http://jaxmice.jax.org/strain/003018.html
